# A short photoperiod alters brain metabolism and cold resistance in *Drosophila melanogaster*

**DOI:** 10.1038/s41598-025-22793-7

**Published:** 2025-10-07

**Authors:** Madhura Sapre, Anna Hovhanyan, Werner Schmitz, Peter Deppisch, Jayati Gera, Martin J. Mueller, Pamela Menegazzi, Agnes Fekete, Charlotte Helfrich-Förster

**Affiliations:** 1 Neurobiology and Genetics, Theodor-Boveri-Institute, University of Würzburg, Am Hubland, 97074 Biocenter, Würzburg, Germany; 2 Molecular Genetics, Theodor-Boveri-Institute, University of Würzburg, Am Hubland, 97074 Biocenter, Würzburg, Germany; 3 Biochemistry and Molecular Biology, Theodor-Boveri-Institute, University of Würzburg, Am Hubland, 97074 Biocenter, Würzburg, Germany; 4 Pharmaceutical Biology, Julius-von-Sachs-Institute, University of Würzburg, Julius-von-Sachs Platz 2, 97084 Biocenter, Würzburg, Germany

**Keywords:** LC-MS based metabolomics, Lipids, Polar metabolites, Photoperiod, Feeding, Activity, Molecular biology, Neuroscience

## Abstract

To survive, animals need to prepare for winter in advance, and this process begins in the brain in response to the shortening of the photoperiod in fall. Here, we demonstrate that exposing adult flies for just 14 days to a short photoperiod at a constant temperature of 20 °C increases their cold resistance and dramatically alters brain metabolism. Such flies have significantly lower levels of monosaccharides, and a lower ATP/AMP ratio in their brains than flies exposed to a long photoperiod, despite being less active and eating more. The levels of storage and structural lipids (triacylglycerols and phospholipids) as well as the number of lipid droplets in the brain increase, suggesting the utilization of glucose for the synthesis of lipids via the citrate shuttle. In addition, during short days, the ratio between the reduced and oxidized forms of glutathione increase, as do detoxification processes and autophagy. This suggests that the brain of short-term flies is less sensitive to oxidative stress and neurodegeneration, which is essential for survival throughout the winter. Overall, our results show that exposure to a short photoperiod has significant metabolic and physiological consequences in the fly brain that serve to prepare for the coming winter.

**Supplementary Information:**

The online version contains supplementary material available at 10.1038/s41598-025-22793-7.

## Introduction

Organisms living in temperate zones are exposed to strong seasonal changes to which they must adapt. Winter poses a problem for organisms for two key reasons: energy and water. Food sources become scarce, humidity decreases, and temperatures fall below the optimal range, which means that the cellular functions that provide energy falter and eventually fail. This is particularly relevant for small organisms such as insects, which are unable to maintain a constant body temperature. When internal temperatures fall below freezing, the water that makes up at least 70% of animal cells freezes, and the organism dies. To avoid suboptimal internal temperatures and dehydration, animals can migrate to warmer regions, enter a dormant state with reduced energy expenditure, and develop resistance to cold and dryness^1^.

To survive, animals must adjust their metabolism long before winter sets in. Those that do not prepare for winter in time will have to deal with severe fitness disadvantages. But how do they recognize that winter is coming? Temperature can fluctuate greatly and is therefore no reliable indicator of the coming season. The most robust cue for assessing the onset of winter is the shortening of day length (photoperiod) in the fall. Therefore, all animals have mechanisms in their brains to measure photoperiod, which communicate with the endocrine system and initiate physiological and metabolic changes when it falls below a critical level, even if ambient temperatures are still pleasant. These mechanisms are called photoperiodic responses and have been well-studied in several mammals and insects^2,3^. They need an internal circadian clock as a reference for measuring day length^4^.

The fly *Drosophila melanogaster* has been successfully used as a model to understand the role of the circadian clock in photoperiodic responses^5–8^. Light and the circadian clock are known to affect metabolite levels in the body and the head of fruit flies^9,10^. However, litte is known about the metabolic responses of the brain to changes in photoperiod. This is surprising, since the brain contains the master circadian clock, processes light information, measures photoperiod and initiates seasonal changes in behavior and in the neuroendocrine system^11,12^. In addition, the brain is one of the most metabolically expensive organs, and this is true for all animals^13^. As an example, in humans, the brain makes up about 2% of body mass but consumes 20% of total energy and this applies during wakefulness and sleep. Consequently, animals have evolved efficient ways to reduce the energy costs of their brain during conditions of food scarcity such as the winter. Some moles even shrink the size of their brain in winter and regrow it in spring^14^. Such extreme effects are extremely unlikely in fruit flies, which only survive the winter once in a dormant state before producing the next generation the following spring. Nevertheless, significant metabolic adaptations to photoperiod shortening are expected. An earlier study showed that flies already increase their cold resistance when reared in short-day conditions compared to flies reared in long-day conditions at the same temperature^5,15^. Since *Drosophila* flies, unlike many other holometabolous insects, do not overwinter as larvae but as adults, and the photoperiod is also measured in the adult stage, we investigated whether exposing adult flies to short-day conditions is sufficient to cause metabolic changes in the brain.

We found that the exposure of adult flies to a short photoperiod is sufficient to increase their cold resistance and to change their brain metabolism, while the metabolism in the whole head and body remain unchanged. In their brains, flies appear to metabolize sugar to synthesize the storage lipids triacylglycerols and structural phospholipids. They increase polyamine levels which enhance autophagy and may extend lifespan, and they appear to become more resistant to cold and oxidative stress. All these changes can be regarded as preparation for the coming winter.

## Materials and methods

### Fly rearing and adult maintenance

Wild-type *Drosophila melanogaster* flies of the strain *CantonS* (WT_cs_) were reared under 12 h:12 h light: dark cycles (LD12:12) at 25 ± 0.2 °C and 60 ± 2% relative humidity on *Drosophila* food consisting of 0.8% agar, 2.2% sugar beet syrup, 8.0% malt extract, 1.8% yeast, 1.0% soy flour, 8.0% corn flour, and 0.3% hydroxybenzoic acid. After eclosion, adult flies were transferred to new fly vials containing the same food and exposed for 14 days either to long (LD16:8) or short (LD8:16) photoperiods, while temperature was kept constant at 20 °± 0.2 °C. In the following experiments, the flies treated in this way are called long- and short-day flies, respectively. All experiments were performed with male flies.

### Locomotor activity recordings

The locomotor activity of 32 flies each was recorded under long (LD16:8) and short (LD8:16) photoperiods in the *Drosophila* Activity Monitor (DAM)-System of TriKinetics (Waltham, MA, USA). Flies were transferred into 5 mm tubes containing food (4% sucrose and 2% agar in water) using CO_2_ anesthesia. Activity was monitored by detecting the disruptions in an infrared light beam at 1-minute intervals. The activity was tracked over a period of about 9 days at 20 °C with 60% relative humidity. Light intensity during the experiments was set to 100 lx.

### Data analysis

To reveal the activity pattern of the flies, activity data were plotted as individual and average actograms using the ImageJ plug-in ActogramJ^16^. Furthermore, individual and average activity profiles were calculated for the two fly groups and overall daily activity levels were determined as described in Schlichting and Helfrich-Förster^17^ for average actograms and average activity profiles (see Figure S1).

### Capillary feeding (CAFE) assay

Feeding was assessed with the CAFÉ assay by two methods. First, food consumption was measured in groups of 15 flies for 2 h after 2 h of starvation, and second, in individual flies for 24 h without starvation. For the first assay 20 groups of flies consisting of 15 long- and short-day flies, each, were transferred to vials without food on day 14, directly after lights-on. After 2 h of starvation, the fly vials were placed at an angle of 45° and two microcapillary pipettes were inserted, one containing 5 µl of normal tap water and the other 5 µl of 5% sucrose solution with blue food dye. The flies could choose freely between the two capillaries. Every 20 min, we recorded how much liquid they had consumed and after 2 h, the total consumed food was determined for all 40 fly groups (in total 300 long- and short-day flies, respectively). An empty vial containing only the two capillaries served as a control for evaporation.

For the second assay, individual flies were confined to 2 ml Eppendorf cups. The top of each cup contained a hole, in which a capillary containing 5 µl of 5% sucrose solution was inserted. There were two more holes on the sides of the tubes for aeration. After 24 h the amount of food consumed by each fly was determined. Like before, an empty vial containing only the capillary was used as a control for evaporation.

### Data analysis

At first, the data were checked for a normal distribution (Kolmogorov-Smirnov test; *p* < 0.008, Shapiro-Wilk test; *p* < 0.03). Since the data deviated significantly from normality, the non-parametric Mann-Whitney-U test was applied for checking statistically significant differences.

### Cold tolerance assay

Cold tolerance was measured by recovery from chill coma (David et al., 1998; Macdonald et al., 2004). Flies from twenty vials, containing 10 long- and short-day flies, each, were transferred to empty fly vials equipped only with water-moistened filter paper. The vials were then stored in darkness on crushed ice at 0 °C for 24 h. After cold exposure, the flies were transferred to 25 °C for recovery. They were immediately individually placed into glass tubes of the DAM TriKinetics system to monitor their activity. The percentage of flies that resumed activity indicating survival of cold exposure was determined. Furthermore, the time until the first movement was measured in flies that survived the treatment.

### Lipid analysis

For the body and head sampling, long- and short-day flies were shock frozen in liquid nitrogen (shortly after lights-on) and stored at −80 °C. Fly heads were separated from the body on ice and three fly heads, and three fly bodies were pooled for each sample. For the brain sampling, long- and short-day flies were quickly killed (shortly after lights-on) by submerging them in 4% paraformaldehyde dissolved in 1X phosphate buffered saline - PBS (Sigma-Aldrich #P5493-1 L, 10X solution diluted to 1X with Millipore water). After 1 h of fixation, the dissection was carried out in 1X PBS. The isolated brains were then transferred into methanol for extraction (Methanol Absolute- Biosolve, ULC/MS-CC/SFC, #13684102). 10 brains were pooled for each sample and stored at −80 °C until analysis. Blight-Dyer extraction was performed as published in Mueller et al.^18^ and lipid profiling of the organic phase with LC-MS was performed in accordance with Schäbler et al.^10^. For lipid fingerprinting, the acquired data used for lipid profiling were pre-processed with Progenesis QI (version 1.0, Waters) and MetaboAnalyst 6.0 was used for statistical analysis^19^. Graphs were generated with R (v4.2.1) via Rstudio (2022-06-23) using ggplot2 package.

### Targeted analysis of water-soluble primary metabolites

Water-soluble metabolites from 10 pooled *Drosophila melanogaster* brains were extracted with 500 µl ice-cold MeOH/H_2_O (80/20, v/v) containing 0.04 µM lamivudine and 4 µM each of D_2_-glucose, D_4_-succinate, D_5_-glycine and^15^N-glutamate (Sigma-Aldrich, St. Louis, USA). After centrifugation, the resulting supernatants were evaporated in a rotary evaporator (Savant, Thermo Fisher Scientific, Waltham, USA). Dry sample extracts were redissolved in 50 µl 5 mM NH_4_OAc in CH_3_CN/H_2_O (50/50, v/v). 20 µl supernatant was transferred to LC-vials. Metabolites were analyzed by LC-MS using the following settings: For LC-MS analysis 3 µl of each sample was applied to a SeQuant ZIC-cHILIC (3 μm particles, 100 × 2.1 mm) (Merck, Darmstadt, Germany). Metabolites were separated with Solvent A, consisting of 5 mM NH_4_OAc in CH_3_CN/H_2_O (40/60, v/v) and solvent B consisting of 5 mM NH_4_OAc in CH_3_CN/H_2_O (95/5, v/v) at a flow rate of 200 µl/min at 45 °C by LC using a DIONEX Ultimate 3000 UHPLC system (Thermo Fisher Scientific, Bremen, Germany). A linear gradient starting after 2 min with 100% solvent B decreasing to 10% solvent B within 23 min, followed by 16 min 10% solvent B and a linear increase to 100% solvent B in 2 min was applied. Recalibration of the column was achieved by 7 min prerun with 100% solvent B before each injection.

All MS-analyses were performed on a high-resolution Q Exactive mass spectrometer equipped with a HESI probe (Thermo Fisher Scientific, Bremen, Germany) in alternating positive and negative full MS mode with a scan range of 69.0–1000 m/z at 70 K resolution and the following ESI source parameters: sheath gas: 30, auxiliary gas: 1, sweep gas: 0, aux gas heater temperature: 120 °C, spray voltage: 3 kV, capillary temperature: 320 °C, S-lens RF level: 50. XIC generation and signal quantitation was performed using TraceFinder™ V 5.1 (Thermo Fisher Scientific, Bremen, Germany) integrating peaks which corresponded to the calculated monoisotopic metabolite masses (MIM +/- H^+^ ± 2 mMU). All analyses were performed in six independent replicates. Graphs were created with R (v4.2.1) via Rstudio (2022-06-23) using ggplot2 package.

### Fluorescent immunohistochemistry and microscopy for lipid droplets

Immunohistochemistry was performed on male flies entrained to long and short photoperiods for 14 days, following the protocol of Schubert et al.^20^, to quantify the number and area of lipid droplets in the brain using BODIPY493/503 (Invitrogen, Cat. No: D3922). Flies were fixed in 4% paraformaldehyde in PBS (1XPBS) with 0.5% Triton X-100 (PBST) for 3.5 h. After three PBS washes, flies were dissected, and the brains were washed twice with PBST. Brains were then incubated overnight at 4 °C with BODIPY493/503, followed by six PBST washes. Finally, brains were mounted on slides using Vectashield (Vector Laboratories, Burlingame, CA, USA) with ~ 150 μm spacers. The images were acquired with a Leica SP8 confocal microscope (Leica Microsystems, Wetzlar, Germany) equipped with hybrid detectors, photon multiplier tube and a white laser for excitation. We used 20-fold glycerol immersion objective (HC PL APO, Leica Microsystems, Wetzlar Germany) and obtained confocal stacks with 2 μm z-step size and 1024 × 512 pixels. The obtained images were analyzed with Fiji ImageJ^21^.

Lipid droplet analysis was performed on the central brain region only. A maximum Z-projection was generated, and noise was reduced using the Despeckle function. Manual thresholding was applied, followed by Watershed to separate overlapping droplets. The Analyze Particles function in ImageJ was used to quantify lipid droplet number and total area. Approximately 13 brains were analyzed per condition.

## Results

### Flies are less active but eat slightly more under short days

**Fig. 1:**
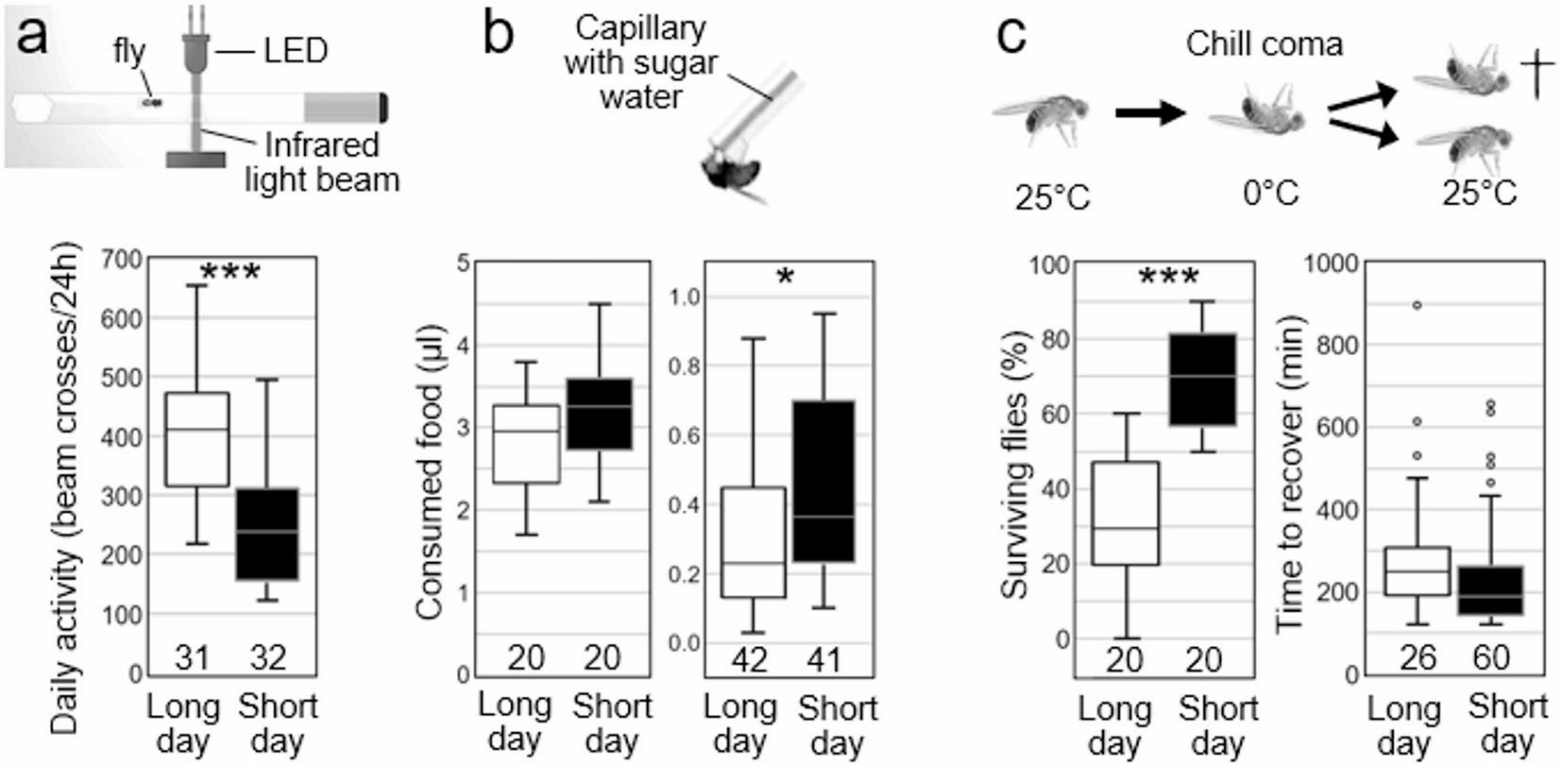
Daily activity, food intake and cold tolerance of long- and short-day flies. **a** Mean daily activity of ~ 30 flies recorded by infrared light beam crosses in TriKinetics monitors (top; details see Fig. S1). **b** Amount of food consumed by 20 groups of 15 flies during a time span of 2 h (left) and single flies throughout 24 h (right) in the capillary feeding (CAFE) assay. **c** Percentage of flies surviving chill coma (left) and time the surviving flies took for recovery (right). The experiment was repeated 20 times with 10 flies each. The time to recover was calculated for all surviving flies. The number of individually tested flies or fly groups is indicated in the bottom of the diagrams. Note that only 26 of the long-day flies survived. Consequently, the time to recover could only be determined in these 26 flies. Asterisks indicate significance (*: *p* < 0.05, **: *p* = 0.01, ***: *p* < 0.001).

Our locomotor activity recordings showed that the flies were significantly less active under short days than under long days (Fig. 1a, S1). This suggests that they would need less energy in short days and consequently should eat less. However, this was not the case. Our capillary feeder assay (CAFE) revealed that short-day flies of the same age appeared hungrier after a short period of food deprivation and consequently ate slightly more than long-day flies (Fig. 1b, left). In addition, they consumed significantly more food during the normal 24-h day than long-day flies (Fig. 1b, right). This suggests that the flies use the extra energy uptake to adapt their metabolism to the coming winter.

### Short days during adulthood increase cold tolerance of the flies

Previous results have revealed that flies reared in short-day conditions increase their cold resistance when compared to flies reared in long-day conditions at the same temperature^5^. Here, we aimed to determine whether a 14-day exposure period to short days during adulthood is enough to increase cold tolerance.

We adopted a simple assay to measure cold tolerance called chill coma recovery, in which flies are exposed for a certain period to cold stress (around 0 °C). Flies are then transferred to room temperature and the percentage of surviving flies, and their recovery time are measured^22,23^. Short-day flies should show a better recovery from chill coma. Indeed, we found that a significantly higher percentage of short-day flies survived an exposure of 24 h to 0 °C (Fig. 1c). We also measured recovery time of the surviving flies by measuring their start of locomotor activity in *Drosophila* activity monitors. We found that the flies took about 4 h to start moving again, and that the long-day flies took slightly longer than the short-day flies. However, this difference was not significant, most likely due to the overall low number of surviving long-day flies.

### Short days alter lipid levels in the brain but not in the body and the head

**Fig. 2:**
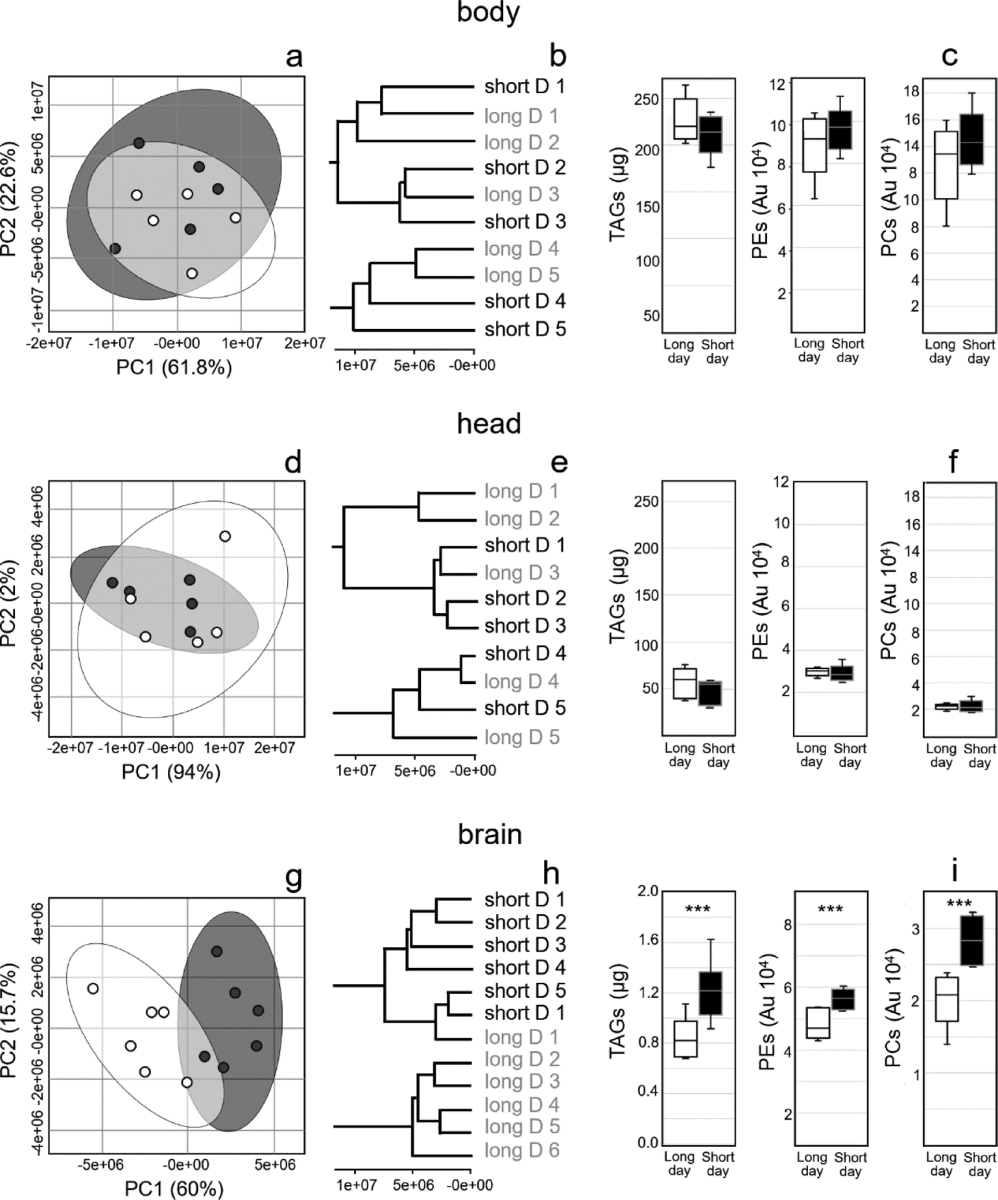
Principal component analysis, clustering and lipid profiling of bodies, heads and brains of long- and short-day flies. Organic phases of all samples (three *Drosophila* bodies and heads and 10 brains were pooled) were analyzed by LC-MS and all detectable lipid features were investigated. The results for long-day flies are shown in white or light gray, and those for short-day flies are shown in black or dark gray. Principal component analysis (a, d, g) and clustering (b, e, h) showed a clear separation of long- and short-day fly samples in brains but not in bodies and heads. The levels of triacylglycerols (TAGs), phosphoethanolamines (PEs), and phosphoglycerocholines (PCs) were determined by lipid profiling (c, f, i) and showed higher levels in the brain of short-day flies compared to long-day flies, but not in the body and head. PC1, PC2: first and second principal components (percent variance in parenthesis), Au: arbitrary unit. The number of replicates was 5 for heads and bodies and 6 for brains. Asterisks indicate significance (*: *p* < 0.05, **: *p* = 0.01, ***: *p* < 0.001).

Higher levels of lipids, particularly triacylglycerols (TAG), have been found in cold-adapted whole flies^24^. Therefore, we first analyzed lipids in the head and body of the fly. Three fly heads and three fly bodies were pooled as one sample, and five replicates were analyzed by LC-MS coupled to electrospray ionization in positive mode. First, we performed a lipid fingerprinting procedure, where all aligned peaks were taken into account to filter out lipid features with significantly different levels. A total of 1337 lipid features (Table S1) were detected in the body (abdomen and thorax) and 1050 in the head (Table S2). Principal component analysis did not show a clear separation between long and short-day flies (Fig. 2a, d), nor did cluster analysis show a clear grouping (Fig. 2b, e). Next, we profiled energy storage lipids (TAGs) and structural lipids (phosphoethanolamines (PEs) and phophoglycerocholines (PCs)) according to Schäbler et al.^10^. The levels of TAGs, PEs and PCs in the body (Fig. 2c) and head (Fig. 2f) were not significantly different between short- and long-day flies. Therefore, we conclude that 14 short days are not sufficient to alter lipid levels in the body and the entire head.

Since the photoperiod is first detected in the brain, which then signals the fat tissue in the head and body to trigger changes, our next step was to analyze the lipidome in isolated brains. Ten brains were pooled for one sample, and six replicates were measured by LC-MS using positive and negative electrospray ionization. First, we applied a metabolite fingerprinting approach to the annotated 875 lipid features (Fig. S2, Table S3). Remarkably, principal component analysis revealed a clear separation between the six long-day and short-day brain samples (Fig. 2g). In addition, unsupervised clustering revealed strong similarities between the six replicates, except for one long-day sample, which was grouped with the short-day samples (Fig. 2h). These results reflect clear differences between the brain lipidome of long- and short-day flies. Finally, lipid profiling showed that the total levels of TAGs, PEs and PCs were significantly higher in the brains of short-day flies compared to those of long-day flies (Fig. 2i).

**Fig. 3:**
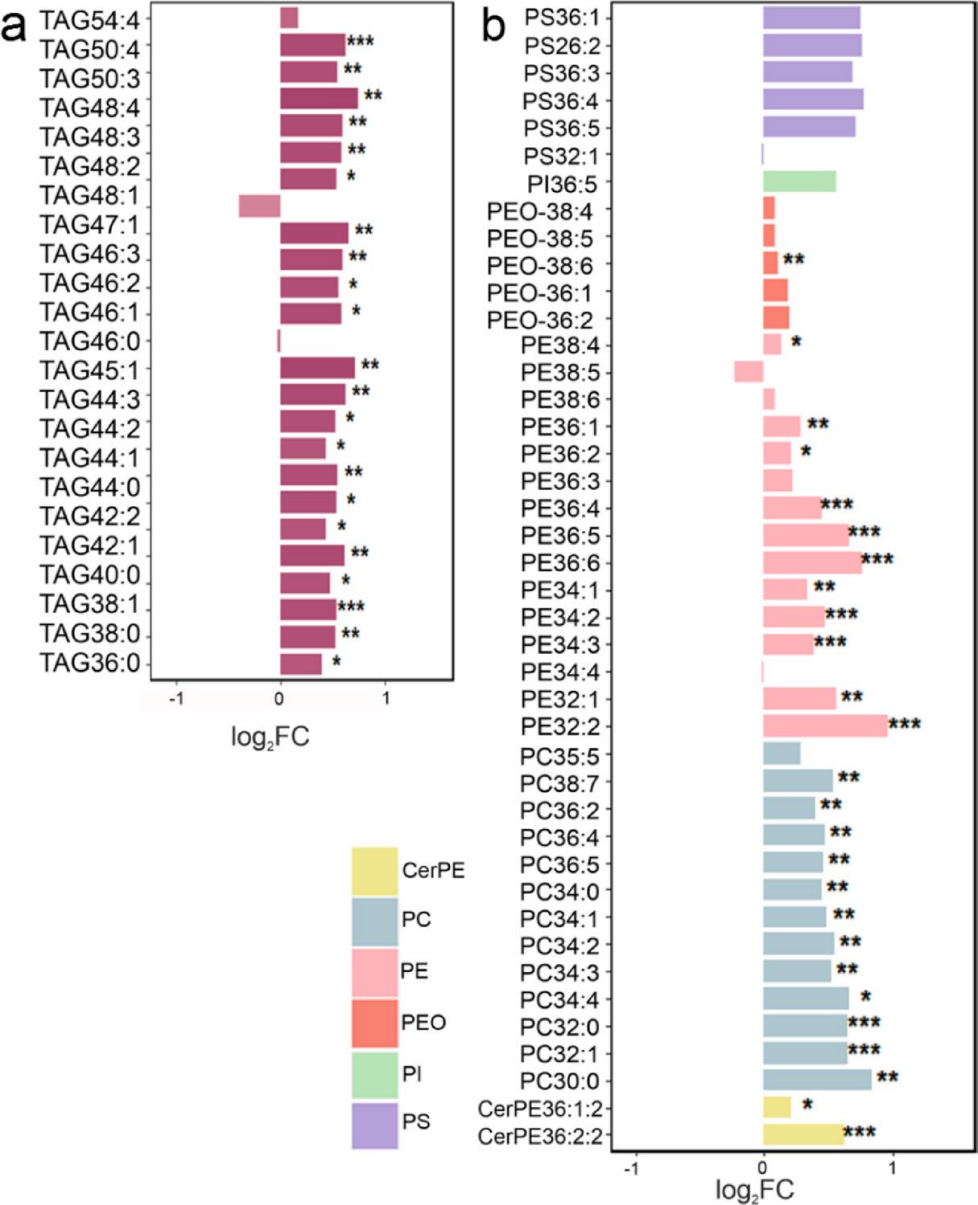
Log2 fold changes in the levels of identified energy storage lipids (a) and structural lipids (b) in the brains of short-day flies in relation to long-day flies (the values in long-day flies were set to 0). Lipid species are defined according to lipid class and acyl chains: the letters indicate the lipid class; the first number is the number of acyl carbons and the last number the number of acyl double bonds. Fold changes are calculated by dividing brain levels averaged from the six replicates in short-day flies by long-day flies. TAG: triacylglycerol, PS: phosphoserine, PEO alkyl-acyl-glycerophosphoethanolamine, PE: phosphoethanolamine, PC: phosphoglycerocholine, CerPE: ceramidophosphoethanolamine. The number of replicates was 5 for heads and bodies and 6 for brains. Asterisks indicate significance (*: *p* < 0.05, **: *p* = 0.01, ***: *p* < 0.001).

To find out which lipid species differ, we used the lipid dataset of Carvalho et al.^25^ and profiled all TAGs, PEs, PCs, ceramidophosphoethanolamines (CerPE), alkyl-acyl-glycerophosphoethanolamines (PEO), phosphoniositols (PI) and phosphoserines (PS) detected in the brains (Fig. 3). We identified 23 energy storage lipids (TAG species), out of which 22 were significantly higher with a fold change of around 1.5 in short-day compared to long-day brains (Fig. 3a). Of structural lipids, we identified 5 PSs, 1 PI, 5 PEOs, 15 PEs, 13 PCs and 2 CerPEs. Except for three species, all structural lipids appeared to have higher levels under short days, and this turned out to be significant for the CerPEs, most PEs, PCs and for one PEO species (Fig. 3b). Lipid profiling results showed that the levels of major triacylglycerols and phospholipids were between 10 and 80% higher in the brains of short-day flies compared to long day flies. This explains well why the principal component analyses separated short-day and long-day samples (Fig. 2g).

The fluidity of the membrane can adapt to changes in environmental temperature, thereby maintaining membrane function homeostasis. One parameter that describes membrane fluidity is the double bond index (DBI), that indicates the average number of double bonds in esterified fatty acids^26^. We did not detect any significant differences in the double bond index of TAGs (long day: 0.403 ± 0.008, short day: 0.411 ± 0.006, *p* = 1.00), PEs (long day: 1.324 ± 0.007, short day: 1.320 ± 0.006, *p* = 1.00) or PCs (long day: 1.088 ± 0.008, short day: 1.081 ± 0.013, *p* = 1.01). Our results suggest that a shortened photoperiod is insufficient to alter the number of double bonds in the esterified fatty acids of energy storage and structural lipids in the brains of *Drosophila melanogaster*.

Our results indicate that photoperiod affects the lipidome in the brain, but not in the fly head and body. Furthermore, 14 short days led to an increase in the levels of energy storage lipids (TAGs) and structural lipids (PEs, PCs, CerPEs, PEOs) in the brain.

### Short days alter the level of water-soluble metabolites involved in brain energy metabolism

To understand which metabolic pathway potentially supports the increasing lipid levels, we compared the water-soluble metabolites in the brains of long- and short-day flies. By LC-MS, we identified 151 metabolites out of which approximately 65 metabolites were significantly different between the two conditions (Table S4; Fig. S3).

**Fig. 4:**
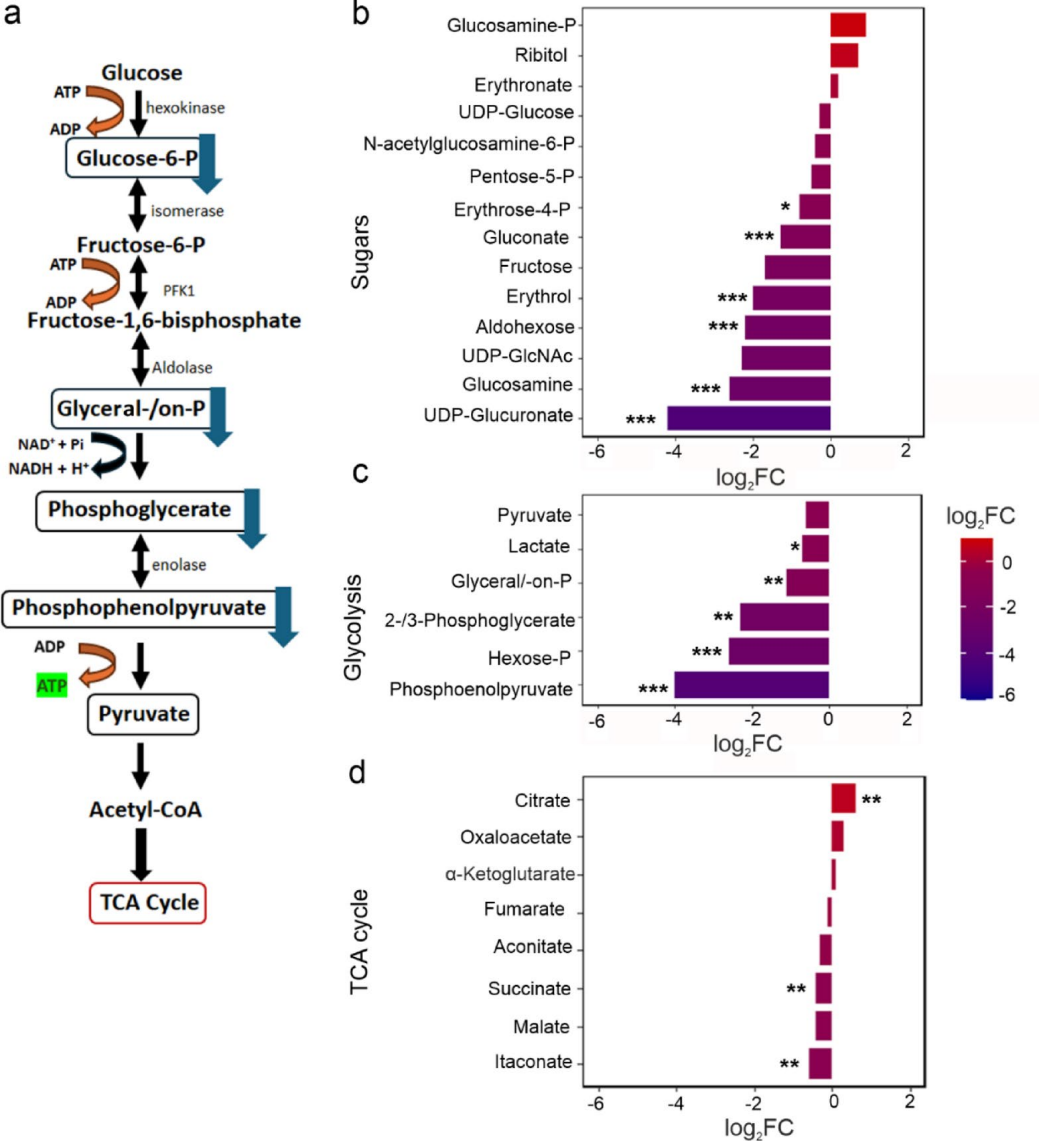
Analysis of water-soluble metabolites related to energy metabolism in the brains of short- and long-day flies. The methanol extract from ten pooled brains was analyzed by LC-MS. The most striking differences are highlighted in panel (a). The Log₂ fold changes in the levels of detected sugars (b), glycolysis intermediates (c) and TCA cycle metabolites (d) were calculated by dividing the mean normalized levels of the short-day flies by the levels of the long-day flies. Asterisks indicate significance: *: *p* < 0.05, **: *p* = 0.01, ***: *p* < 0.001; (*n* = 6).

**Fig. 5:**
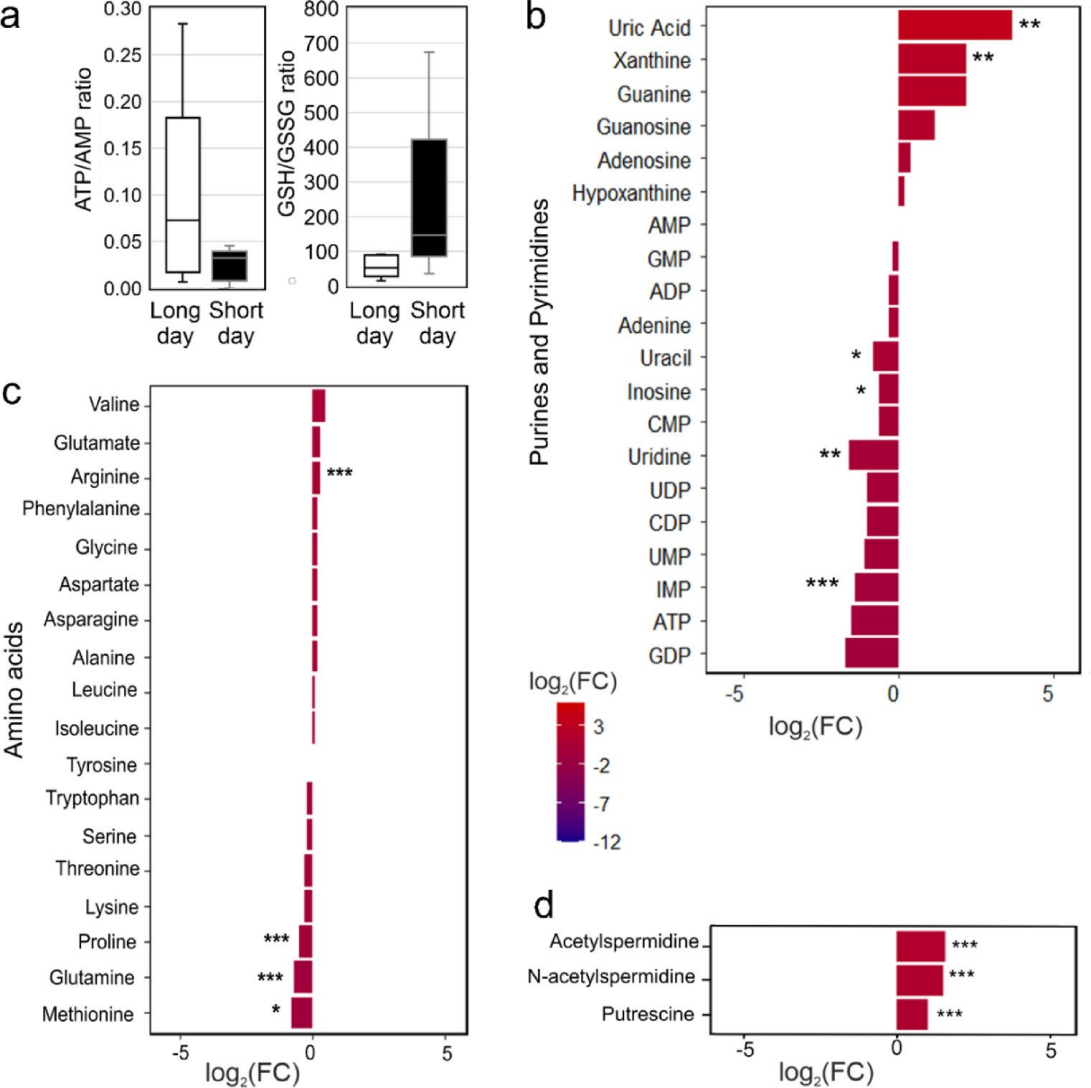
Analysis of water-soluble metabolites related to energy and redox state, as well as nucleotide and amino acid metabolism in the brains of short- and long-day flies. (a) Ratios of ATP/AMP and the reduced/oxidized forms of glutathione (GSH/GSSG). (b -d) Log₂ fold changes in the levels of detected nucleotides (b), amino acids (c) and polyamines (d). The methanol extract of ten pooled brains was analyzed by LC-MS and the log₂ fold changes were calculated as indicated in Fig. 4. Asterisks indicate significance: * *p* < 0.05, ** *p* = 0.01, *** *p* < 0.001; for the GSH/GSSG ratio the significance was *p* = 0.05; (*n* = 6).

We observed lower levels in monosaccharides (sugars) in short-day flies as compared to long-day flies (Fig. 4b). Since this reduction in sugar levels is not caused by decreased feeding (Fig. 1b), it likely results from increased breakdown of glucose via glycolysis. This glycolytic process might supply energy in the form of ATP or generate acetyl-CoA which can be further used for the synthesis of lipids and other metabolites. Indeed, we found that the glycolysis intermediates phosphoglycerate and phosphoenolpyruvate were lower in short day flies, while pyruvate levels were not significantly reduced (Fig. 4c). This could speak for a rapid conversion of glucose to pyruvate in glycolysis (Fig. 4a) and a subsequent utilization of pyruvate for the synthesis of acetyl-CoA. Notably, ATP/AMP ratios had the tendency to be lower in short-day brains (Fig. 5a) suggesting that the ATP produced in glycolysis was used up for anabolic processes (e.g., ATP citrate lyase for lipid synthesis).

Acetyl-CoA, produced by mitochondrial pyruvate dehydrogenase, enters the tricarboxylic acid (TCA) cycle via citrate synthase, transferring the acetyl moiety to oxaloacetate, thereby generating citrate. We detected 8 TCA cycle intermediates and indeed found a slight increase only in citrate and oxaloacetate in short-day flies (Fig. 4d). On the other hand, the amounts of oxaloacetate precursors (malate and fumarate), as well as products, generated from citrate within the TCA cycle (a-ketoglutarate and succinate) did not significantly increase, suggesting that citrate is not being used for gaining energy in the TCA cycle, but instead is shuttled from mitochondria to the cytosol for the synthesis of other compounds such as fatty acids (Figs. 3 and 4).

As already mentioned, the ratio between ATP and AMP tended to be lower in short-day brains (Fig. 5a) suggesting that the energy gained in glycolysis is immediately consumed for anabolic processes. In addition, the ratio between the reduced and oxidized forms of glutathione (GSH/GSSG) tended to be higher in the brains of short-day flies compared to those of long-day flies (Fig. 5a).

The nucleotide analysis revealed lower levels of several purines and pyrimidines in short-day as compared to long-day flies (Fig. 5b). In particular, the mono- and di-phosphates of the purine nucleotides inosine, adenosine and guanosine (IMP, AMP, ADP, GMP and GDP) and the pyrimidine nucleotides uracil and cytosine (UMP, UDP, and CMP) were lower under short days (Fig. 5b). In addition, purine degradation appears to be enhanced under short days, since the end products of purine breakdown, uric acid and xanthine are significantly elevated under such conditions (Fig. 5b). We also found slightly higher arginine levels in the brains of short-day flies (Fig. 5c). Arginine is produced in the urea cycle that serves the detoxification of ammonia during the breakdown of amino acids in the mitochondria. Otherwise, most amino acid levels were comparable between short and long days. Only the amino acids proline, glutamine and methionine were slightly but significantly lower under short-day conditions (Fig. 5c). Overall, our results suggest increased degradation of nucleotides and a minor influence on amino acid levels under short days.

Another interesting finding of our analysis was the increase in polyamines levels (putrescine, spermidine) in short days (Fig. 5d). Polyamines are low-molecular‐mass, ubiquitous polycations that are well documented for combating senescence and stress, a role that is attributed to them for their cell‐membrane‐stabilizing, free‐radical–scavenging, and acid‐neutralizing properties^27^. In plants, polyamines are known as cryoprotectants that increase the accumulation of osmolytes, regulate redox homeostasis, stabilize membranes, and regulate gene expression to survive cold stress^28,29^. It is not known whether polyamines have also cryoprotective functions in animals, but if so, this can explain the higher chill coma survival rate of short-day flies. In animals, the crucial role of polyamines in autophagy, which removes unnecessary components from the brain, reduces neurodegeneration, and extends lifespan, is well documented^30^. Flies may need all the changes discussed, caused by increased polyamine levels in the brain, to survive the winter.

### Short days increase the number of lipid droplets in the brain

**Fig. 6:**
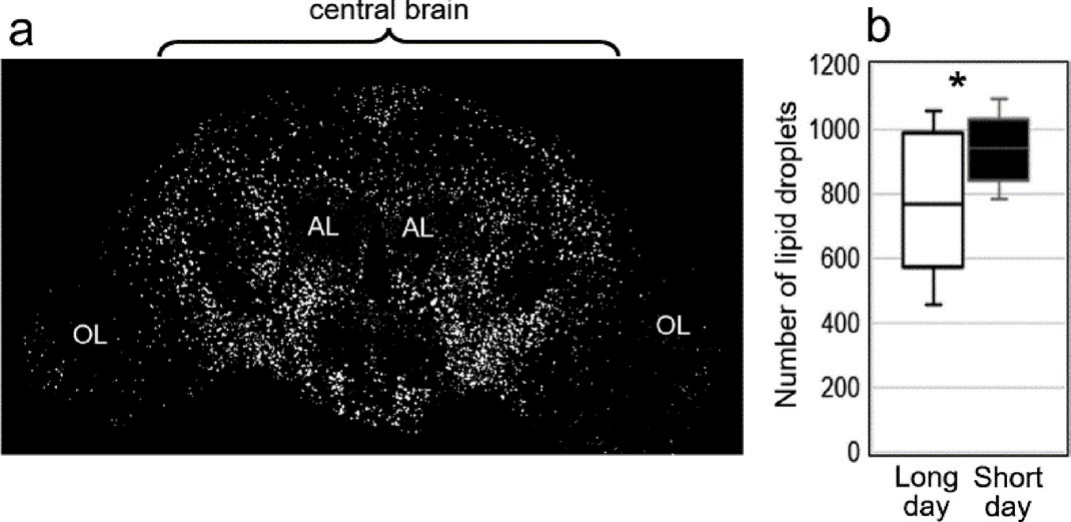
Lipid droplets in the brain of long- and short-day flies. **a** Overlay of 40 confocal stacks of the anterior brain stained with “BODIPY493/503” for lipid droplets. AL: antennal lobes; OL optic lobes. **b** Number of lipid droplets in the central brain of long- and short-day flies. *: *p* < 0.05 (*n* = 13).

Lipid droplets are intracellular organelles responsible for energy storage, consisting of a hydrophobic core—primarily triacylglycerols (TAGs)—enclosed in a phospholipid monolayer coated with proteins. The increase in both storage lipids (TAGs) and structural lipids like PEs, PCs and PSs that we observed (Fig. 2i) suggest a potential impact of photoperiod on the abundance of lipid droplets in the fly brain. To investigate this, we stained and quantified the number of lipid droplets in the brains of short- and long-day flies. Lipid droplets were predominantly localized to the cell bodies of the central brain and to a lesser extent in the optic lobes (Fig. 6a). We, therefore, focused our analysis on the central brain, and quantified the number of lipid droplets present. We found that short-day flies exhibited a significantly higher number of lipid droplets (Fig. 6b).

In summary, our results suggest that day length can significantly modulate metabolic pathways in the brain. As a main effect, sugars are degraded under short days, most probably for the synthesis of fatty acids. In addition, it appears that ammonia detoxification, autophagy and resistance to oxidative stress are increased under short-day conditions, which might increase cold resistance and lengthen lifespan under short-day conditions. All of this can be seen as preparation of the brain for the coming winter. Depending on the genetic background and latitude of origin, reproductive flies live for around 40 to 80 days under laboratory conditions at 25 °C^31,32^ while dormant flies can survive for several months (throughout the winter) under semi-natural conditions in field cages^33^, until environmental conditions become favorable again (in spring).

## Discussion

Photoperiodic responses are known in many organisms living in temperate zones and exposed to strong seasonal changes. Preparing in advance for the coming winter is essential for survival, which is why organisms change their physiology and metabolism in response to the shortening of the photoperiod in autumn. Adult fruit flies have a lifespan of a few weeks in summer, but in autumn their lifespan increases dramatically so that they survive the entire winter without food in a dormant state^33^. To make this possible, they increase their cold and overall stress resistance, fill their lipid stores and lower their general metabolism^34^. These changes happen in response to the decreasing photoperiod and temperature in autumn^35^. Although fruit flies of the species *Drosophila melanogaster* cannot be regarded as typical photoperiodic animals, they clearly respond to a shortening of daylength in autumn^6,36^. Previous studies showed that *D. melanogaster* reared for one generation under short photoperiods but at a constant temperature of 20 °C, increase their cold resistance^5,15^. Here, we report that in adult flies, 14 days of exposure to short days (8 h of light and 16 h of darkness) at a temperature of 20 °C is already sufficient to increase cold resistance.

Furthermore, we show that this exposure significantly alters lipid metabolism in the brain, but not in the head and body. This suggests that the brain is the first organ to respond to seasonal changes and confirms that it is the place where the length of the day is measured with the help of the circadian clock in mammals and insects^11,37^. As soon as the daylength falls below a certain threshold, this information is transmitted via several pathways to the neuroendocrine system of the brain, which alters neurohormonal signaling to the body (Helfrich-Förster, 2024). Another reason why metabolic changes are first observed in the brain is probably because of its high energy requirements^38,39^. The brain requires a lot of energy even when the animal is resting, sleeping or hibernating, while the skeletal muscles have almost no energy requirements at these times. Therefore, this energy requirement must be met first. We assume that the fatty tissue (fat body) in the head and body of the flies will respond with a similar increase in lipids after a few more short days, but this still needs to be tested.

### Preparation for the winter needs energy

Our results show that flies eat more under short-day conditions but are less active than under long-days, suggesting that they use excess energy for anabolic processes. This is in line with previous studies that have measured the oxygen consumption of adult short-day and long-day flies. Flies that were kept under short-day conditions (8 h of light and 16 h of darkness) for three days and flies that had already been reared under short-day conditions consumed significantly more oxygen than flies kept under long-day conditions during the same period^40,41^.

### Flies appear to use excess energy for lipid synthesis

**Fig. 7:**
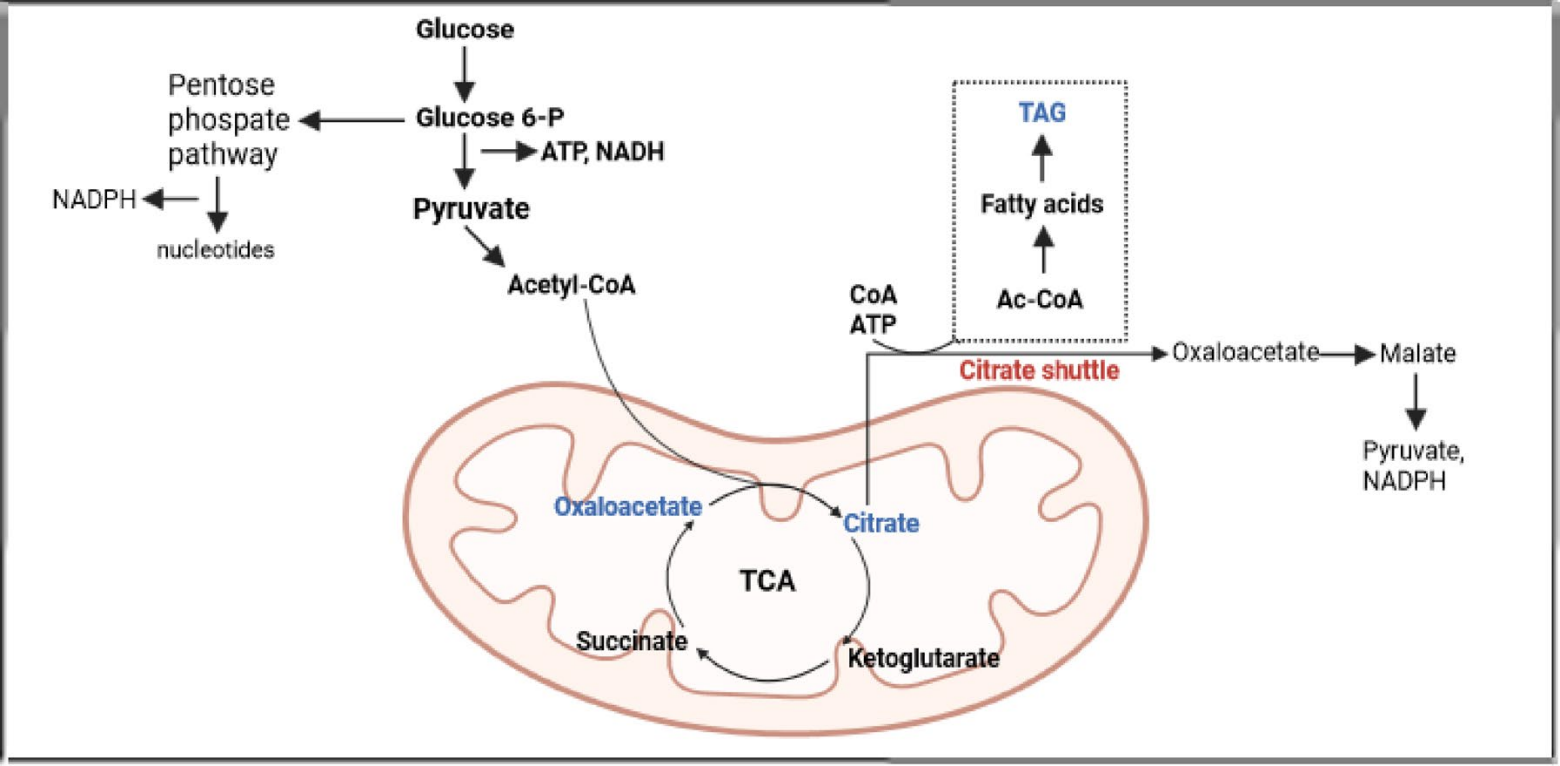
Schematic model: Metabolic substrates from glycolysis, pentose-phosphate pathway and TCA cycle are utilized for lipid synthesis via citrate shuttle.

Short-day flies appear to increase the levels of all major lipid classes in their brains at the expense of nucleotide and protein synthesis. We propose the following metabolic shift under short days: glycolytic and pentose phosphate pathway intermediates decrease, along with most TCA cycle metabolites—except for citrate and oxaloacetate. Due to the decreased levels of ribose, *de novo* nucleotide biosynthesis might be low under short-day conditions as visible in low levels of nucleotide intermediates. But we also detected high levels of xanthine and uric acid, the products of purine degradation in short-day flies, indicating a shift toward catabolic purine turnover. Based on our observations, we suggest that under short photoperiod, acetyl-CoA and ATP generated through glycolysis are utilized by mitochondrial TCA cycle to produce citrate, which is subsequently transported via the citrate shuttle to the cytosol for fatty acid synthesis (Fig. 7).

Remarkably, all major lipid classes including storage (TAGs) and membrane lipids (PEs, PCs, PEOs, PSs, CerPEs) are elevated in the brain of short-day fly brains. While the synthesis and accumulation of storage lipids (TAGs) can be understood as immediate preparation for winter, the synthesis of membrane lipids appears less clear – at least at the first glance. Lipids are stored in droplets surrounded by a phospholipid monolayer. In short-day flies, there was a notable increase in lipid droplet accumulation, particularly in the central brain. This rise in lipid droplets may also contribute to an overall increase in membrane phospholipids. Additionally, these phospholipids also contribute to apoptosis- particularly, phosphatidylserine (PS) which enables the fusion of exocytotic vesicles with plasma membrane and serves as a signal for the recognition and clearance of apoptotic cells. In *Drosophila*, PE is a key phospholipid essential for maintaining the stability of the mitochondrial inner membrane. It is synthesized from PCs and PSs by the genes *Pss* and *Pisd*. Mutations in *Pss* lead to neurodegeneration, mitochondrial dysfunction and elevated reactive oxygen species (ROS) levels^42^. According to these findings, we assume that the elevated levels of PSs, PCs, and PEs under short days, might help in the maintenance of mitochondrial integrity, reduce oxidative stress, and support normal cellular function.

### Short days appear to reduce oxidative stress and increase detoxification and neuroprotection

Since short-day flies have to survive the entire winter, they need to protect their brains from neurodegeneration. Oxidative stress, characterized by an imbalance between ROS and antioxidant defenses, plays a significant role in the progression of neurodegeneration. The brain is one of organs especially vulnerable to the effects of ROS because of its high oxygen demand and its abundance of peroxidation-susceptible lipid cells. In humans, previous studies have demonstrated that oxidative stress plays a central role in a common pathophysiology of neurodegenerative diseases such as Alzheimer’s and Parkinson’s disease^43,44^. We found a high ratio between reduced glutathione (GSH) and oxidized glutathione (GSSG) in the brains of short-day flies. GSH is a key antioxidant that enables a high degree of antioxidant defense minimizing oxidative damage. In healthy cells, the GSH/GSSG ratio is typically around 100:1 ^45^, indicating a reducing environment. This means that most of the glutathione is in its reduced form (GSH), which is crucial for its antioxidant functions. In short-day flies we found a GSH/GSSG ratio up to 400:1 (Fig. 5a), which can be regarded as very high (under conditions of oxidative stress it can drop to 10:1 or even 1:1). Another antioxidant is uric acid, the product of purine metabolism, which can scavenge ROS and is also elevated in short-day flies. GSH and uric acid may work together to mitigate damage caused by oxidative stress. In addition, we detected elevated levels of polyamines in short-day flies. Spermidine is a natural polyamine that induces autophagy, an evolutionarily conserved recycling process of waste products in cells in eukaryotes that prolongs the lifespan of all animals studied to date, including *Drosophila*^46^.

Overall, our study suggests that photoperiod alone is sufficient to initiate the physiological changes necessary for seasonal adaptation, while temperature may amplify these responses. We propose a model in which short-day flies utilize metabolic resources specifically for energy production and storage in the brain (Fig. 7).

### Relevance of our study for humans

Many of the metabolic adaptation processes in *Drosophila melanogaster* are regulated by molecular signaling pathways that are highly conserved in mammals including humans. For example, spermidine promotes cell clearance, maintains mitochondrial function, and improves memory performance in aging humans^46–48^, and oxidative stress plays a similar role in neurodegeneration in the human brain as it does in the brain of fruit flies^44,45^. It is also well known that humans respond to photoperiodic changes^49,50^. When the photoperiod becomes shorter in autumn, human metabolism changes and people tend to eat more^51^, just as we have shown here for fruit flies. It is likely that the shortening of the photoperiod in autumn can also lead to metabolic changes in the human brain. Thus, the photoperiod-controlled metabolic reprogramming in the fly brain could serve as a valuable model for important aspects of seasonal physiology, circadian regulation, and stress resilience in the human brain. It is obvious that the modern human lifestyle under artificial conditions of eternal summer is the most extreme example of disconnection from the natural seasons that might make humans susceptible to increased morbidity and mortality^52^. Understanding how environmental light stimuli influence central energy distribution and neuroprotection mechanisms could open new therapeutic strategies for treating cognitive aging processes and metabolic brain disorders in humans.

## Supplementary Information

Below is the link to the electronic supplementary material.

Supplementary Material 1

Supplementary Material 2

Supplementary Material 3

Supplementary Material 4

Supplementary Material 5

## Data Availability

All metabolomic data generated or analyzed during this study are included in this published article (and its Supplementary Information files).
