# Supplementary material for: A short photoperiod alters brain metabolism and cold resistance in Drosophila melanogaster
Source: Sci Rep. 2025 Oct 7;15:35035. doi: 10.1038/s41598-025-22793-7 (PMC12504615; doi:10.1038/s41598-025-22793-7)
Supplement: Supplementary file 1 — Supplementary Material 1 [file 41598_2025_22793_MOESM1_ESM.pdf]

A short photoperiod alters brain metabolism and cold resistance in *Drosophila melanogaster*

Madhura Sapre, Anna Hovhanyan, Werner Schmitz, Peter Deppisch, Jayati Gera, Martin J Mueller, Pamela Menegazzi, Agnes Fekete, Charlotte Helfrich-Förster

Supplementary material

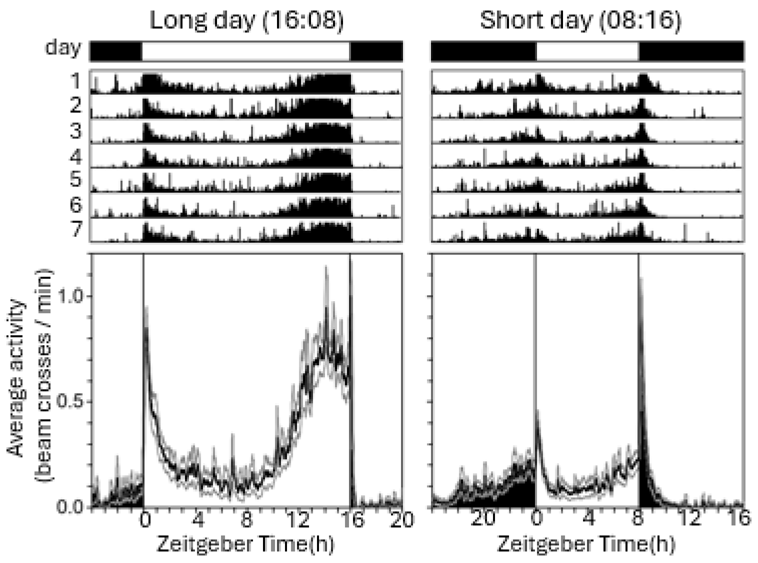

Figure S1: Average actograms and average activity profiles of ~30 male WT_{CS} flies under long and short days. The white and black bars on top of the actograms indicate the light program. The flies were recorded for seven consecutive day and the average activity profiles depict the flies' mean activity of all seven days (± standard error of the mean).

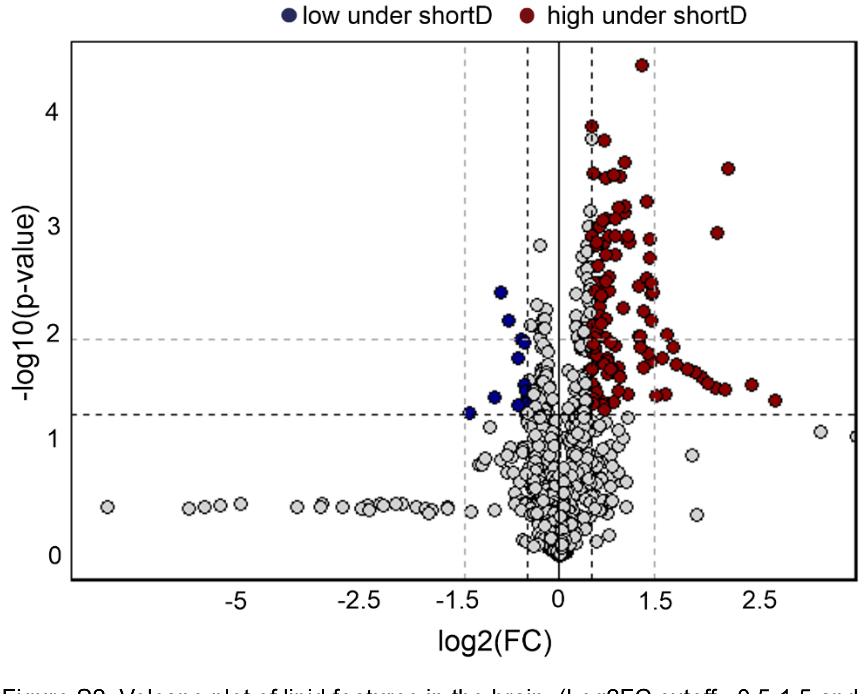

Figure S2: Volcano plot of lipid features in the brain. (Log2FC cutoff= 0.5-1.5 and p-value=0.05-0.01)

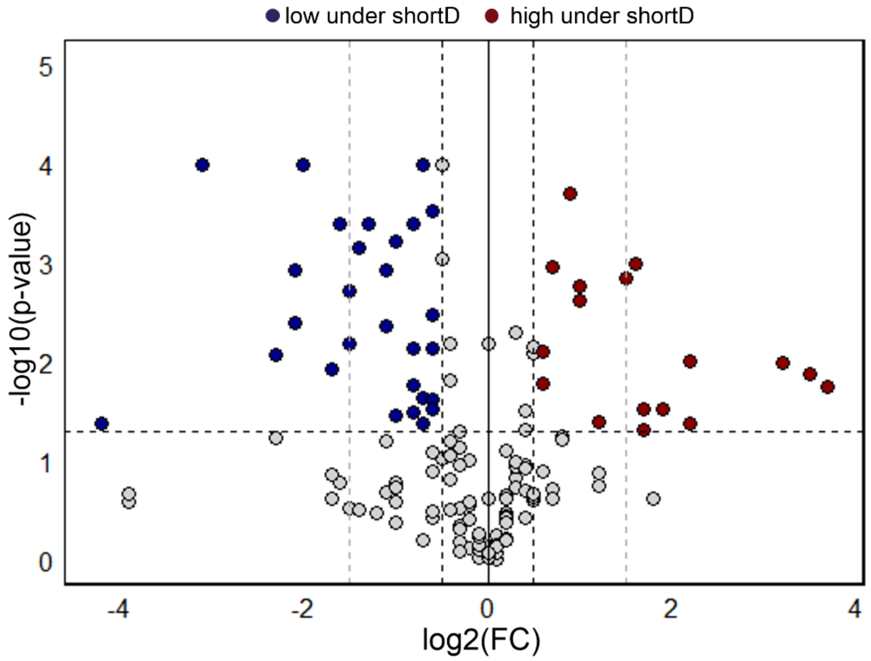

Figure S3: Volcano plot of primary metabolites in the brain. (Log_2FC cutoff =1 and p-value= <0.05)

Supplementary Tables are added as Excel Files.

Tables S1 to S3 show the lipid metabolites found in the body (Table S1), head (Table S2) and brain (Table S3) of short- and long-day flies. In all three tables, the metabolites characterized by profiling are listed first, followed by the unknown metabolites identified by untargeted analysis. The body and head extracts stem from the same three flies and were analyzed together, while the brain extracts stem from ten different flies, from which the brains were dissected. Five replicates were taken for the body and head extracts, and six for the brain extracts. Normalized peak areas from mass spectrometry are shown for all replicates in the final five or six columns of each table.

Please note that the lipidome measured in brains does not necessarily overlap with that measured in heads. This is because the head contains several other tissues, such as the compound eyes, the fat body, air sacs, the trachea and the mouthparts, including the esophagus and its associated muscles. It also contains the antennae and many sensory bristles. The fat body constitutes a significant proportion of the head as it serves as a nutrient store and a detoxification organ (the insect's liver). Therefore, we assume that the lipidome measured in heads primarily represents that of the fat body. Consequently, the lipidome measured in heads only overlaps marginally with that measured in brains. In general, fewer lipid species were detected in the head. This may be for the following reasons: (1) Pooling three heads may not provide sufficient material to detect low-level lipid features in the brain. (2) Due to the hard cuticle, homogenization of the heads results in lower extraction efficiency than homogenization of dissected brains, leading to fewer extracted brain lipids and a lower concentration of those extracted.

Tables S4 shows water-soluble metabolites identified in the brains of short- and long-day flies.
